# Mechanisms of HIF-driven immunosuppression in tumour microenvironment

**DOI:** 10.1186/s43046-023-00186-z

**Published:** 2023-08-30

**Authors:** Shinjini Bandopadhyay, Somi Patranabis

**Affiliations:** Amity Institute of Biotechnology, Amity University, Kolkata, West Bengal India

**Keywords:** Tumour immunogenicity, Tumour microenvironment, Hypoxia, HIF, Immunotherapy

## Abstract

Hypoxia arises due to insufficient oxygen delivery to rapidly proliferating tumour cells that outpace the available blood supply. It is a characteristic feature of most solid tumour microenvironments and plays a critical role in regulating anti-tumour immunity, enhancing tumoral heterogeneity, and promoting therapeutic resistance and poor clinical outcomes. Hypoxia-inducible factors (HIFs) are the major hypoxia-responsive transcription factors that are activated under low oxygenation conditions and have been identified to drive multifunctional roles in tumour immune evasion. The HIF signalling network serves as an attractive target for targeted therapeutic approaches. This review aims to provide a comprehensive overview of the most crucial mechanisms by which HIF controls the expression of immunosuppressive molecules and immune checkpoints, disrupts cancer immunogenicity, and induces immunotherapeutic resistance.

## Background

### Tumour immune microenvironment (TIME)

Tumour cells do not act in isolation but exist in a complex and dynamic ecosystem with their microenvironment. Malignant cancer cells of solid tumours are associated with non-malignant host stroma, consisting of extracellular matrix (ECM) components, fibroblast cells, mesenchymal cells, blood and lymph vasculature, and tumour-infiltrating immune cells, cytokines, and chemokines [1]. The immune cells in the tumour microenvironment (TME) are of both innate and adaptive type and primarily include tumour-associated macrophages (TAMs), neutrophils, dendritic cells (DCs), myeloid-derived suppressor cells (MDSC), natural killer (NK) cells, and CD4 + and CD8 + T lymphocytes and B lymphocytes, along with several extracellular immune factors [2, 3]. All immune components of the TME constitute the tumour immune microenvironment (TIME), and the varied functions and spatial organization of immune cells in the tumour microenvironment influence tumour progression, anti-tumour immune responses, and the efficacy of immunotherapeutic interventions. Extensive research from recent decades has illustrated the crucial roles of the host immune system in controlling anti-tumour and pro-regulatory immune response through cancer immune surveillance and tumour interaction. For instance, higher populations of MDSCs and TAMs in the TIME have been observed to promote tumour progression, whereas the increased recruitment of cytotoxic T lymphocytes has been associated with improved anti-tumour immune response and better prognosis [4]. Alterations in cancer cells and the surrounding stromal tissue due to environmental stresses like hypoxia can modify the immune response to tumours [3].

**Fig. 1:**
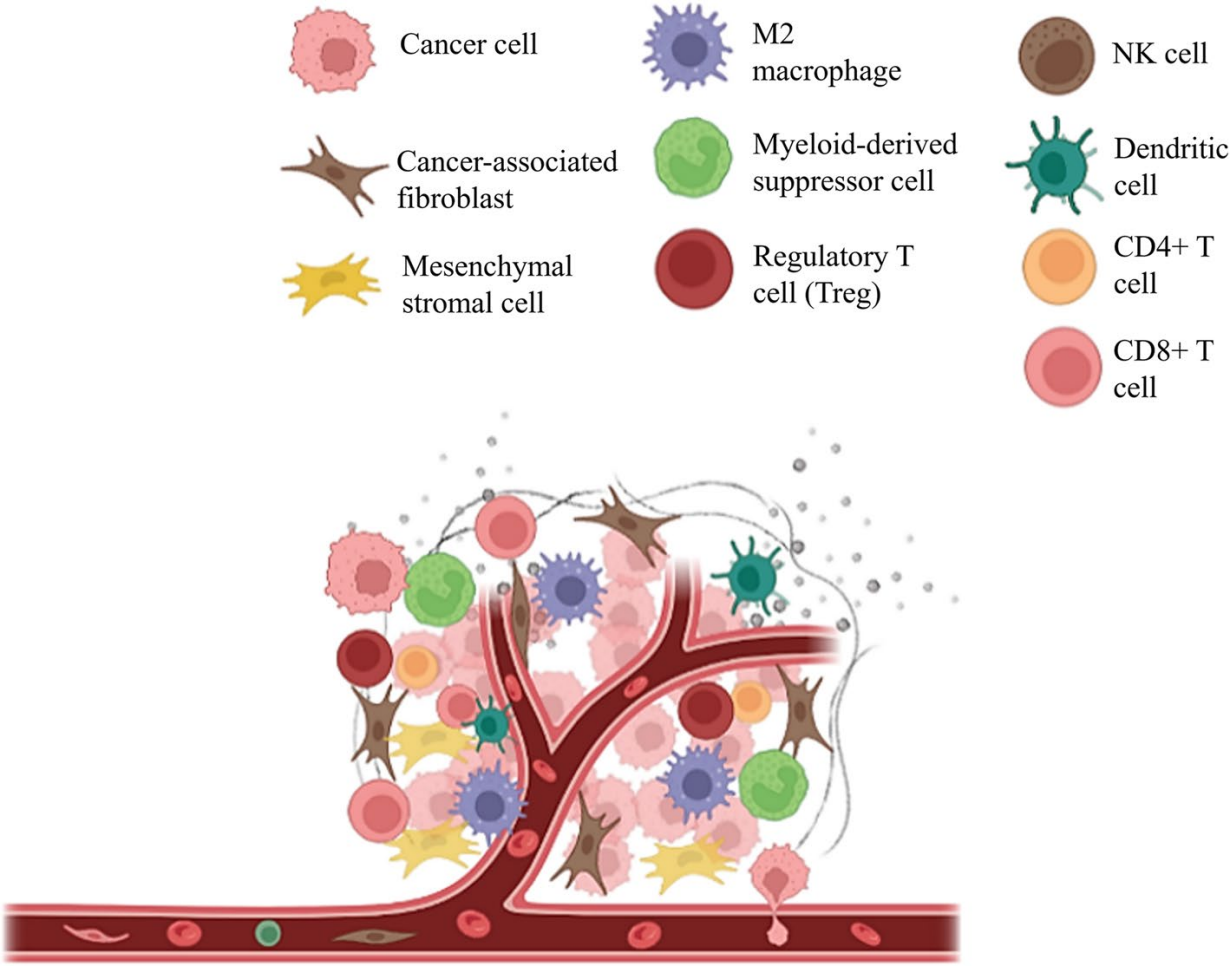
Tumour immune microenvironment (TIME)

The immune cells of the TIME (Fig. 1) can be broadly classified into immunosuppressive (tumour-promoting) and immune effector (tumour-antagonizing) cells [5]. The tumour-antagonizing immune cells mainly consist of effector T cells, including CD8 + cytotoxic T lymphocytes (CTLs) and effector CD4 + T cells, NK cells, dendritic cells (DCs), M1-polarized macrophages, and N1-polarized neutrophils. The CTLs are the major subset of lymphocytes for killing the cancer cells; when presented with tumour antigens from DCs, CD8 + T cells can be induced into effector CD8 + CTLs with cytotoxic capacity. NK cells are also an important subset of tumour-antagonizing immune cells with a similar function, with respect to CD8 + T cells, and are attracted to cancer tissues under the guidance of chemokines secreted by DCs. They attack tumour cells by releasing perforin and granzymes to induce apoptosis. DCs mainly function as professional antigen-presenting cells (APCs), which can present antigens and provide costimulatory signals for T-cell activation and interact with NK cells and B cells [5]. TAMs are the primary tumour-infiltrating immune cell types in the TIME [6]. They are generally categorized into classical activated M1 macrophages, which typically have anti-tumour functions, as well as alternatively activated M2 macrophages, which are immunosuppressive. The latter exhibit pro-tumour functions, including inhibition of T-cell-mediated anti-tumour immune response. Both M1 and M2 macrophages have a high degree of plasticity and can be converted into each other upon tumour microenvironment changes like hypoxic stress [7].

Other tumour-promoting immune cells mainly consist of regulatory T cells (Tregs) and MDSCs. Tregs are a specialized subset of CD4 + T cells identified by the expression of the FOXP3 gene, and they induce tumour tolerance by the production of TGF-β and suppression of effector T cells [8]. Myeloid-derived suppressor cells (MDSCs) are a heterogeneous population of immature myeloid cells, which can be classified into two main subtypes: polymorphonuclear (PMN-MDSC) and monocytic (M-MDSC). Besides directly repressing DCs, NK cells, and T cells, and promoting immune tolerance, MDSCs also contribute to angiogenesis and metastases [9, 10].

### Hypoxic TME and HIFs

During tumour development and progression, cancer and stromal cells often have restricted access to nutrients and oxygen. Most solid tumours consist of regions that are permanently or transiently subjected to hypoxia, owing to aberrant vascularisation and poor blood supply [11].

The hypoxic TME is defined as a condition of poor oxygenation where partial O_2_ pressure drops below 10 mmHg [12]. Hypoxia arises from the imbalance of increased oxygen consumption by rapidly proliferating tumour cells and the available blood supply. Inadequate oxygen supply triggers new blood vessel formation or angiogenesis, but the distribution of the newly developed tumour vasculature network is irregular and characterized by diffusion limits, leakiness, and malformation. This leads to pockets of different oxygen tensions in the TME and contributes to the heterogeneity of the spatial architecture of the cells [13, 14]. At the molecular level, the response and adaption of tumour cells to the hypoxic TME are largely mediated by the hypoxia-inducible factor (HIF) family of transcription factors. HIFs are heterodimeric helix-loop-helix proteins consisting of an O_2_-sensitive α-subunit (HIF-1α, HIF-2α, and HIF-3α) and a constitutively expressed β-subunit (HIF-1β) [15]. HIF-1α and HIF-2α have crucial roles in the positive hypoxic response and are the best studied, whereas HIF-3α is considered a negative regulator [16, 17]. Most of the research on hypoxic regulation of the tumour microenvironment highlights the functions of HIF-1 which has a diverse range of effects. Cellular sensing of oxygen status regulates the stabilization of the HIF-1 protein under conditions of sufficient and insufficient oxygenation. In normoxic conditions, the conserved proline residues of HIF-1α undergo hydroxylation by prolyl hydroxylases (PHDs) and are bound by the Von Hippel-Lindau tumour suppressor protein (pVHL). This catalyses ubiquitination-dependent proteasomal degradation. Another factor regulating HIF-α in an oxygen-dependent manner is the factor inhibiting HIF1 (FIH1). Asparagine hydroxylation of HIF1-α (and sometimes of HIF2-α) driven by FIH1 prevents the interaction of HIF1 with its transcriptional co-activator factors, p300 and CBP, further inhibiting HIF1 transcriptional activity. However, in deprivation of oxygen or hypoxia, the oxygen-dependent PHDs and FIH cannot function, allowing HIF-1α accumulation and translocation to the nucleus, where it heterodimerizes with HIF-1β to form active HIF-1. Active HIF-1 recognizes and binds to specific promoter regions of various genes known as hypoxia-response elements (HREs) to drive the transcriptional activation of hundreds of target genes and pathways (Fig. 1) [17].

**Fig. 2:**
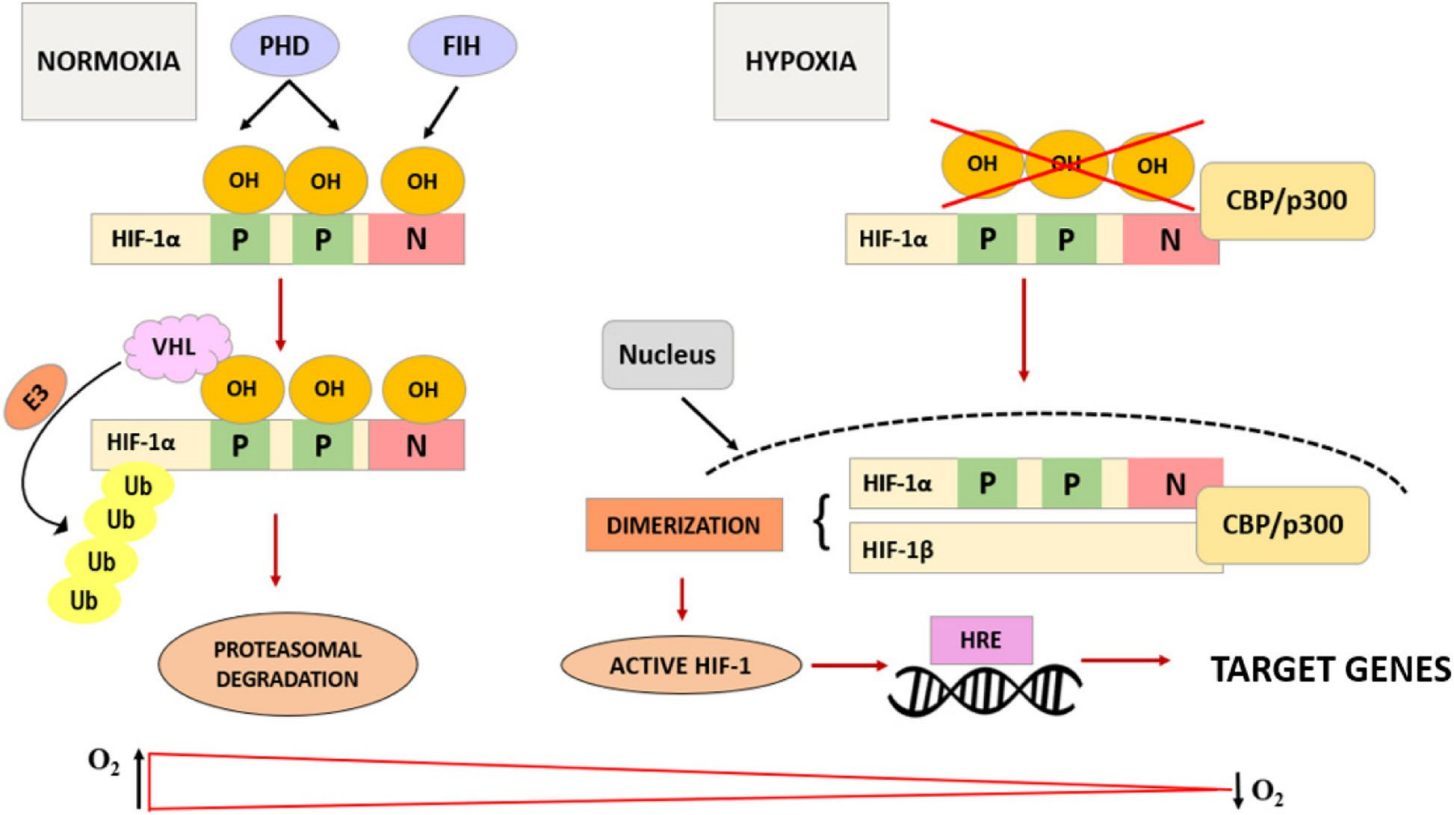
Cellular oxygen sensing and regulation of HIF-1 in normoxia vs. hypoxia

HIF-dependent signalling can promote the adaptation and selection of both cancer and stromal cells to the surrounding environment to foster changes that favour cancer progression (Fig. 2). Cancer cells show a distinct reprogrammed metabolic phenotype characterized by increased glycolysis and preferential production of lactate, as opposed to mitochondrial oxidative phosphorylation. This phenomenon is called the Warburg effect or aerobic glycolysis [18]. HIF activity switches the cell metabolism of the TME toward the glycolytic pathway, thus increasing glucose consumption and pyruvate, lactate, and H + production. While HIF-1 plays a major role in glycolytic gene regulation, HIF-2 is mainly involved in pluripotent stem cell maintenance and angiogenesis, resulting in enhanced pro-tumourigenic phenotype. HIF-1α is mainly expressed during acute hypoxia (in the first 24 h) in all tissues, while HIF-2α is stabilized during chronic hypoxia (later stages of prolonged hypoxic conditions) with tissue-specific expression. Although the expression of HIF-3α is detectable in a variety of human cancer cell lines, it is comparatively less investigated. HIF-3α lacks a transactivation domain, suggesting that this isoform possesses a suppressive effect toward the other HIF isoforms [17].

HIF-1α and HIF-2α are structurally similar except for their transactivation domain. HIF-1α generally binds HREs close to gene promoters, while HIF-2α targets transcriptional enhancers. Thus, although both have overlapping targets, several are unique target genes. The isoform specificity influencing the outcome of the transcriptional programmes has been investigated in several studies and has been found to vary depending upon the cell type and severity and duration of hypoxia [17, 19].

Despite having several nonredundant functions, there is no evidence in recent literature suggesting differential regulation of tumour immunity by HIF-1 and HIF-2 or under acute and chronic hypoxic conditions. Whether or not the different HIF isoforms have contrasting effects in mediating immunosuppression in the TME remains to be explored.

### HIF-mediated immunosuppression in cancer

Hypoxic regions in solid tumours are infiltrated with several immunosuppressive cells, such as TAMs, MDSCs, and Treg cells, which limit access to NK cells and CD8 + T cells [20]. HIF-1 signalling plays a significant role in regulating the immunosuppressive nature of TMEs through a variety of mechanisms which are discussed here. By acting upon tumour-infiltrating immune cells, enhancing the recruitment of immune-tolerant cells, and directing the transcriptional activation of immunosuppressive factors, hypoxia has been identified to dampen the activities of effector cells like cytotoxic T lymphocytes (CTLs), NK cells, and DC cells. Hypoxia upregulates immunosuppressive regulatory T cells, MDSCs, and TAMs, promotes the secretion of immune-suppressing cytokines and chemokines, and interferes with antigen-presenting cells [8].

#### Regulation of immunosuppressive cells in TIME

Research from recent decades shows that HIF-1α inhibits anti-tumour immunity by modifying the TIME cells and promoting the release of cross-immunosuppressive factors. Hypoxia drives the immunosuppressive function of Treg cells and contributes to immune tolerance by the direct binding of HIF-1 to the FOXP3 promoter region in CD4+ T cells. This promotes the transcription of Foxp3 in a transforming growth factor beta (TGF-β)-dependent mechanism to stimulate their differentiation into Treg cells [8, 21]. There is prior evidence of HIF-1α-induced TGF-β activation in tumours, such as breast cancer, where HIF-1α was recognized as a positive upstream regulator of the TGF-β1/SMAD3 pathway, leading to tumour progression and poor clinical outcome [22]. In gastric cancer, hypoxia promotes TGF-β1 secretion from tumour cells and subsequently enhances Foxp3 expression of T cells [23]. Crosstalk between HIF-1α and TGF-β is observed to drive tumour progression and aggressiveness through a combined synergistic effect. This is observed in a number of solid cancers like renal cell carcinoma and prostate cancer, where both engage in a positive interaction loop [24]. One of the features of TGF-beta1 is that it undergoes a functional change from suppression of cancer cell proliferation in early stages of cancer growth to inhibition of T-cell-mediated anti-cancer immunity in late-stage tumours [25, 26]. Studies have attempted to understand and pinpoint the underlying molecular mechanism behind these opposing functions. An interesting observation by Huang et al. in a recent study, suggests a correlation between the levels of HIF-1α expression with the TGF-beta ‘switch’ [27]. Their work confirmed that the regulatory role of TGF-β in non-small cell lung cancer (NSCLC) is affected by a change in oxygen tension. However, early-stage normoxic tumours with small sizes and relatively sufficient blood supply do not show HIF stabilization. With increasing tumour volume and the development of hypoxia, HIF-1α expression increases and binds with SMAD3 to form a transcription complex. This is the main trigger to dysregulate the early-stage function of TGF-β by altering the binding partners of SMAD3. Such an event causes TGF-β to lose its normal inhibitory effect on the proto-oncogene c-Myc and disrupts TGF-β-mediated regulation of p15/p21 proteins, thus showing a completely contrasting pro-tumourigenic effect, as opposed to its initial tumour inhibitory action. Besides being involved in Treg differentiation, TGF-β also plays an important role in the metabolic reprogramming of hypoxic tumours. Although it significantly inhibits glycolysis under normoxia, it facilitates the Warburg effect in hypoxia after the release of c-Myc inhibition. Huang et al. demonstrated how HIF-1α can change the regulatory effect of TGF-β on glucose metabolism at advanced tumour stages via the HIF-1α-SMAD3 complex [27]. This TME shift to glycolysis can, in turn, potentiate immunosuppressive events by further modulation of tumour immunity, such as by decreasing the lysosomal degradation of HIF-2 [28]. HIF-1α can also promote the recruitment of Treg cells to the TME, by stimulating the overexpression of immunosuppressive CC chemokine ligands 22 and 28, as seen in ovarian and hepatocellular carcinomas [29–31]. CCL28 binds to its receptor CCR10 to effectively recruit CCR10+ Treg cells to the tumour site, thus suppressing the functions of effector T cells. In basal-like breast cancer, Treg recruitment has been associated with hypoxia-induced CXCR4 upregulation in Tregs [8, 32].

One of the most widely studied consequences of tumoral hypoxia is the formation of new blood vessels (angiogenesis) to sustain the oxygen-deprived cancer cells through HIF-driven activation of proangiogenic genes, especially vascular endothelial growth factor (VEGF) [33, 34]. Elevated VEGF levels are associated with poor clinical outcomes in several tumours because, in addition to angiogenic effects, VEGF has an important role in the suppression of anti-tumour immunity. VEGF inhibits the maturation of DCs and subsequent activation of CD8+ cytotoxic T lymphocytes (CTLs). This induces an immunosuppressive TME by strongly activating Treg cells, TAMs, and MDSCs. Moreover, tumour-derived VEGF, interleukin (IL)-10, and prostaglandin E3 cooperatively induce Fas ligand expression in endothelial cells, leading to exhaustion and killing of CD8+ CTLs [35]. Under TME hypoxia, VEGF is transcriptionally activated by HIF-1α, which supports the escape of tumour cells from immune surveillance. It does so by recruiting TAMs, Tregs, and MDSCs into the TME, either directly or through upregulation of VEGF [36]. The VEGF/HIF pathway is now being targeted in several cancers, and functional crosstalk among TAMs, Tregs, and MDSCs in the hypoxic TME have been strongly associated with HIF-induced VEGF production [35, 37]. Additionally, hypoxia and TGF-β are major factors that can increase VEGF production both independently and in cooperation. Hypoxic induction of TGF-β expression produces a feedback loop, which increases VEGFA production, providing another therapeutic target [24].

High concentrations of lactic acid production in the TME due to anaerobic metabolism can also enhance VEGF expression [38]. VEGF and TGF-β are two important TME factors, which are favourable for the differentiation of macrophages into immunosuppressive M2 TAMs. TAMs coexpress a mixture of both tumour-type-specific M1 and M2 markers, and TAM polarization can be strongly influenced by their spatial arrangement in tumours. In hypoxic niches, M1 TAMs can polarise into M2-like proangiogenic and immunosuppressive phenotypes. Various factors enriched in these regions, including prostaglandin E2, TGF-β, VEGF, IL-4, IL-6, and ROS, facilitate this polarisation. Additionally, hypoxic tumour cells produce lactic acid which induces M2-associated genes, to further promote the transition of M1 to M2 TAMs [39, 40].

Hypoxia also dramatically alters the function of MDSCs in the TME and redirects their differentiation toward TAMs via HIF-1α [41]. In hepatocellular carcinoma, HIF-1α is reported to promote the migration and differentiation of TAMs from immature myeloid cells via VEGF exposure [42]. TAMs can also secrete MMP7 in hypoxic tumour regions, which can cleave Fas-ligand from the neighbouring cells and protect cancer cells from Fas-ligand-mediated killing by T cells and NK cells [43].

Apart from triggering the differentiation of MDSCs into M2 TAMs, hypoxia is also involved in accumulating and maintaining the function of MDSCs [9]. HIF can induce the recruitment of CX3CR1-expressing MDSCs by activating the transcription of CCL26 in tumour cells and can increase MDSC-mediated T-cell repression by directly binding to the HRE located in the promoter of microRNA (miR)-210 [44].

Besides recruiting immunosuppressive cells, HIF-1α can also negatively regulate functions of effector cells, by directly interfering with T-cell receptor signal transduction [45]. Hypoxia enhances the synthesis of CD39 and CD73 enzymes, which are important factors in the immunosuppressive mechanism, involving adenosine production in the TME. Adenosine is produced by hydrolysis of tumour cell-derived ATP and ADP and released in the TME through membrane channels, cell death, or granular components. Interaction of free adenosine with the adenosine A2A receptors (A2AR) on T cells that are transcriptionally induced by HIF-1 and HIF-2 in hypoxic TMEs leads to the accumulation of immunosuppressive intracellular cAMP and subsequent inhibition of T-cell proliferation and cytotoxicity [17, 46]. High Treg infiltration and TGF-β expression by HIF-1α hinder NK cell functions [47]. Zhang et al. demonstrated that increased HIF-2α levels could suppress natural killer T (NK-T)-cell activation by downregulating the expression of Fas-ligands and simultaneously inducing A2AR expression [48]. Exposure of tumour cells to hypoxia inhibits specific CTL-mediated lysis by a mechanism involving nuclear translocation of HIF-1α, phosphorylation of STAT3, and VEGF secretion by tumour cells [49].

Other diverse pathways of direct and indirect action of HIF on a variety of effector immune cells are described further.

#### Expression of immune checkpoints

HIF-1α induces the upregulation of several immune checkpoint ligands on tumour cells and tumour-associated cells, including PD-L1 and HLA-G.

Tumour cells and other antigen-presenting cells, like tumour-infiltrating myeloid cells, express programmed death ligand (PD-L1 or PD-L2) in response to environmental cues like cytokines, hypoxia, or growth factors. The interaction of the PD ligands with the programmed death-1 (PD-1) receptor induces T-cell apoptosis, T-cell exhaustion, and overall suppression of T-cell-mediated anticancer immunity. Inhibiting the PD‐1/PD ligand (PD‐L1, PD‐L2) binding thus reinvigorates immune rejection of tumour cells, which is a process known as immune checkpoint inhibition and can be achieved clinically using monoclonal antibodies [50]. PD-L1 expression in the TIME exploits the immune tolerance system to facilitate tumour survival and immune escape. This can be controlled by several signalling pathways. Hypoxia can upregulate the expression of PD-L1 in malignant cells and MDSCs via HIF-1α and HIF-2α. Noman M. Z. et al. reported a significant increase in PD-L1 expression in tumour-infiltrating MDSCs induced by the binding of HIF-1 to the HRE4-binding site in the PD-L1 proximal promoter. Furthermore, the blockade of PD-L1 under hypoxia enhanced MDSC-mediated T-cell activation [51]. In glioma cells, PD-L1 has been identified as a direct transcriptional target of HIF-1α, by direct binding to the PD-L1 promoter region, leading to high PD-L1 expression in hypoxic regions. Based on these results, Ding et al. hypothesized the potential success of blocking HIF-1 signalling, together with PD-L1 blockade using checkpoint inhibition therapy. Combining checkpoint inhibition with HIF blockade has indeed shown improved immunotherapeutic results in hypoxic tumours. For instance, targeting the HIF-1α/PD-L1 axis in hypoxic murine breast cancer cells has been observed to restore the activity of tumour-infiltrating lymphocytes [50].

Another indirect pathway of HIF1-mediated PD-L1 signalling has been recently studied in cutaneous melanoma. Single-cell RNA-seq analysis of human cutaneous melanoma datasets revealed a high correlation of HIF-1 and PD-L1 signalling. Although no direct HIF-1-mediated transcriptional control of PD-L1 was observed upon further investigation, HIF-1 was seen to enhance IFNγ-induced PD-L1 mRNA expression in an indirect hypoxic regulation. The authors also state from their studies that HIF-1 alone may be insufficient to induce PD-L1 expression and may cooperate with other factors to trigger its upregulation [52]. High expression levels of PD-L2 in response to hypoxic signals have also been implicated in HIF-1-mediated regulation, as seen in malignant phaeochromocytomas and paragangliomas. This interaction has been associated with TME inflammation and immunosuppression [53, 54]. However, the exact molecular mechanism behind the HIF-1/PD-L2 crosstalk remains to be investigated [54].

The immune checkpoint HLA-G is a non-classical MHC-I molecule, which normally regulates physiological immune tolerance at the foetal-maternal interface, to prevent immunological rejection of the foetus. It is also constitutively expressed in immune-privileged sites such as thymus and cornea [55]. HLA-G is abnormally expressed in tumour tissues, where its immunosuppressive roles are exploited to facilitate tumour immune escape and induction of immune cell tolerance and exhaustion [55], although its functional importance in dictating clinical prognosis has been contested in a recent study on HLA-G expression in carcinomas [56]. Regardless, HLA-G and its receptors have been identified as important immunotherapeutic targets, due to its broad range of immunosuppressive effects. This acts in combination with other crucial immune checkpoints like PD-1, to disarm anti-tumour immunity, by affecting both innate and adaptive immune responses [57]. HIF-1α activates HLA-G expression through HREs, located on the HLA-G promoter region and at exon 2; Yaghi, Layale et al. identified for the first time an HLA-G transcriptional target site of HIF-1 in their 2016 study. They detected HIF-1 to directly activate HLA-G gene expression through an HRE located in coding exon 2 by inducing acute hypoxic stress in glioma cells placed in hypoxia-mimicking conditions [58]. A comprehensive review by Garziera, Marica et al. examined several studies reporting HIF-1-induced HLA-G expression in different human cancer cell lines. They reported that opposite HLA-G transcriptional activities are observed when different tumour types are exposed to hypoxic stress, whereby HIF-1 acts as a negative or positive regulator of HLA-G, depending on the type of cell line (HLA-G− or HLA-G+). In hypoxic conditions, HLA-G− cell lines transcribed HLA-G without any observed translation of the protein. However, HLA-G-positive cell lines showed reduced HLA-G transcriptional activity and protein level, which may be linked to epigenetic regulation through methylation/demethylation of the HLA-G promoter and post-transcriptional regulations [59].

#### Disruption of tumour immunogenicity

Effective tumour immunogenicity relies on the antigen processing and cell surface antigen presentation abilities of tumour cells, to sufficiently induce strong anti-tumour immune responses. Antigenic presentation to DCs and macrophages activates tumour-associated antigens (TAAs)-specific T cells to produce specific cytokines and engage NK cells and B cells in cytotoxic functions to eliminate the tumour [60]. CD8+ T cells are the primary mediators of anticancer immunity. They recognize antigenic peptides presented by the major histocompatibility complex (MHC in vertebrates, also known as human leukocyte antigen or HLA in humans) class I molecules on tumour cells, to become stimulated and kill cancer cells [61]. Poorly immunogenic or non-immunogenic tumours fail to trigger immune responses or generate weak T-cell responses, allowing tumour cells to escape immune surveillance [62]. Since cell surface presentation of peptides by MHC class 1 molecules is critical for the activation of CD8+ T-cell-mediated adaptive immune responses [60], interference in antigen presentation and subsequent disruption of tumour immunogenicity, due to reduced or lost MHC expression, significantly compromises immune recognition and anti-tumour immunity [63–65]. The oxygen tension developed in a hypoxic TME acts as an extrinsic signal, modulating the antigen presentation in tumour cells, and has been identified to suppress MHC class 1 expression levels via HIF-mediated transcriptional regulation [66]. In vitro and in vivo analysis of this mechanism in mouse fibrosarcoma, as well as in human renal cell carcinoma (RCC), evidenced the action of both HIF-1α and 2α in downregulating MHC class I expression, by affecting the transcription of MHC class I heavy chains. The siRNA-mediated knockdown of HIF-1α was also observed to prevent hypoxia-mediated downregulation of both surface expression and transcripts of MHC class I in mouse fibrosarcoma. Whether these functions of HIF, in regulating MHC class I transcription, are by direct promoter binding or through indirect pathways involving secondary downstream molecules is yet to be understood [66, 67]. The same study by Sethumadhavan et al. further explored the effects of HIF regulation on the expression of TAP1, TAP2, LMP2, and LMP7, which are critical components of the antigen processing machinery and are involved in determining the density of cell surface peptide-MHC complexes. LMP2 and LMP7 are important subunits of the proteasomal machinery, responsible for generating peptide antigens, destined to be loaded onto MHC class I. The transporters TAP1 and TAP2 are essential for transporting those processed antigens to the endoplasmic reticulum for complexing with MHC class I. Abnormalities in any or all of these proteins have been identified to result in defective antigen processing and presentation in many cancer cell lines [68]. Furthermore, TAPs and LMPs have been identified as potential targets of the VHL/HIF-1 signalling pathway, whereby they are transcriptionally downregulated in an HIF-dependent manner [69]. In the chosen study, hypoxic conditions were seen to repress the expression of TAP1/2 and LMP7 in mouse fibrosarcoma, both in vitro and in vivo, further exacerbating the disruption of tumour immunogenicity [66].

Several genes of the antigen presentation pathway, including MHC class I and II genes, have been determined as VHL targets [69]. An interesting observation of the data obtained by Sethumadhavan et al. was the effects of hyperoxia in mediating the upregulation of MHC class I expression. Exposure to a hyperoxic environment reversed tumour tissue hypoxia and counteracted the HIF-induced MHC class I downregulation. It also enhanced the expression of MHC, TAPs, and LMPs compared to normoxia. This suggests the existence of an oxygen-regulated mechanism, possibly independent of hypoxia and HIF degradation, which can be further targeted for improving immunotherapeutic efficacy [66].

Another set of molecules that play important roles in antigen presentation are the major histocompatibility complex class I chain-related (MIC) genes MICA and MICB, which encode cell membrane proteins that act as ligands for NK cells. Although these proteins are rarely expressed by normal cells, they are observed in a variety of malignancies and are recognized by the immune system [70]. NK cells express activating receptors, such as NKG2D, which bind to stress-induced ligands MICA and MICB, expressed by the tumour cells. The binding of NKG2D with MICA/B stimulates NK cell cytolytic activity and tumour cell elimination. Hence, downregulated levels or loss of surface MIC proteins promote tumour immune evasion [71]. This interaction of NK cells with MICA and MICB proteins can be suppressed in the hypoxic TME in two ways. Firstly, tumour hypoxia can encourage the shedding of MICA from the tumour cell surface by the HIF-1α-mediated activation of the transmembrane enzyme ADAM10 [72]. The detachment of MICA from cell surfaces depends on a disintegrin and a transmembrane metalloproteinase 10 (ADAM10). The HIF-1α-dependent upregulation of ADAM10 leads to the loss of tumour surface MICA and subsequently suppresses NK recognition and immune response [53]. Alternatively, the expression of HIF-1α in NK cells in a hypoxic TIME can also impair their ability to upregulate the surface expression of the major activating NK-cell receptors, including NKG2D, thereby restricting the NK/MIC signalling [73]. The decreased expression of the activating NKG2D receptors and the induction of granzyme B and intracellular perforin by hypoxia have been described in haematopoietic tumours [39].

**Fig. 3:**
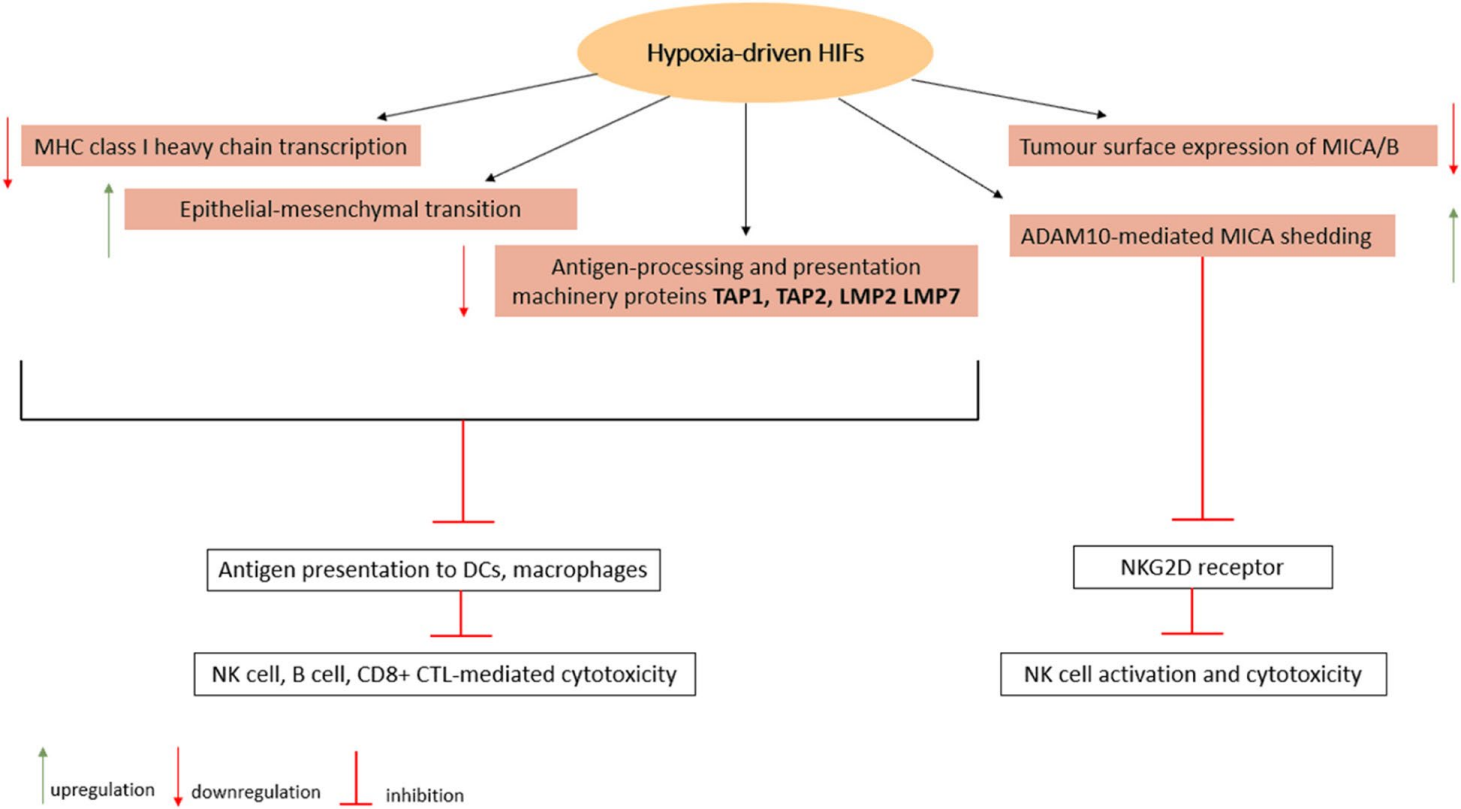
HIF-mediated regulation of tumour immunogenicity

With regard to immune evasion, as a result of low tumour immunogenicity, the processing and presentation of antigens can also be manipulated by tumour cells that are undergoing epithelial-mesenchymal transition (EMT). The acquisition of a mesenchymal phenotype by carcinoma cells is associated with a reduction in tumour immunogenicity, because of defective antigen presentation. Downregulation of antigen processing/presentation components and MHC class I expression, in a variety of epithelial cancers, has been observed because of EMT activation, leading to a reduction in immune recognition by CD8+ T cells against tumours [74, 75]. HIF is identified as a potent regulator of the EMT programme, with abundant experimental evidence of HIF-mediated transcriptional activation of EMT-inducive signalling pathways and EMT-associated genes [76, 77]. Furthermore, HIF-mediated regulation of EMT events has been observed both in hypoxic and normoxic conditions [77, 78]. Due to the multifaceted roles of HIF in promoting EMT, and subsequently facilitating further alterations in effective antigen presentation, the signalling networks of HIF and EMT-related molecules serve as another important therapeutic target to investigate in this context. Figure 3 describes the various mechanisms of HIF-mediated interference with tumour immunogenicity.

Finally, the release of immunomodulatory molecules is another important factor in antigen presentation. Immunogenic cell death or ICD is a form of cell death characterized by the chronic release of such immunomodulatory molecules, known as damage-associated molecular patterns (DAMPs). The number of DAMPs in the TME correlates strongly with the immunogenicity of a tumour [15]. The exposure, active secretion, or passive release of numerous ICD-associated DAMPs like surface-exposed calreticulin (CALR) as well as secreted ATP, annexin A1 (ANXA1), type I interferon, and high-mobility group box 1 (HMGB1) enhances tumour antigenic stimuli to trigger potent anti-tumour immune responses. These can be recognized by both the innate and adaptive immune systems, for the lysis of cancer cells [79]. Interestingly, despite the numerous hypoxic alterations in the TIME, resulting in disrupted or defective tumour immunogenicity, low oxygenation conditions have been demonstrated to promote tumour cell death through ICD. This happens by enhancing the cell surface exposure of calreticulin in an ER stress-dependent manner [80]. Although the involvement of HIF activity in this study has not been characterized, this finding calls for further investigation and molecular studies, on the role of hypoxia in cancer immunity. This is needed to unravel the nature of pro-tumourigenic as well as anti-tumourigenic roles of hypoxic TMEs in tumour immune response. While hypoxia is generally associated with worse prognostic outcomes, selective studies like this have demonstrated that oxygen-deprived TMEs can create the environment necessary for cell death via ICD.

#### Hypoxia-associated autophagy

Hypoxia is associated with the cell homeostatic regulators, such as autophagy and the unfolded protein response (UPR). Autophagy is a catabolic pathway that degrades cytosolic components, including proteins and organelles. The autophagosomes capture the cytosolic materials, and fuse with lysosomes, to mediate their degradation [61]. Such degradation provides nutrients required to maintain cellular functions under stress conditions, such as hypoxia [12]. During tumour progression, autophagy acts as a survival mechanism, which is induced by different stressors, including hypoxia. Autophagy promotes tumour cell survival and protects them from anti-tumour cytotoxic immune responses by NK cells and T lymphocytes. This happens through mechanisms including impairment of antigen presentation and promotion of checkpoint expression [53, 81].

Hypoxia-induced autophagy can occur in an HIF-dependent or independent manner to impair the susceptibility of tumour cell to immune cell attack [12] It is an important regulator of the innate and adaptive anti-tumour immunity. Core autophagic machinery components have been identified as HIF-1 targets, such as BCL2 and adenovirus E1B 19 kDa-interacting protein 3 (BNIP3), BNIP3-like (BNIP3L)/NIX, Beclin 1, and phosphatidylinositol 3 kinase catalytic subunit type 3 (PIK3C3). HIF-1 can also regulate autophagy by altering glucose metabolism, through molecules like glucose transporters-1/3 (GLUT1/3), hexokinases (HK1/2), lactate dehydrogenase (LDHA), phosphoglycerate kinase 1 (PGK1), pyruvate dehydrogenase kinase 1(PDK1), enolase 1 (ENO1), and 6-phosphofructo-2-kinase/fructose-2,6-bisphosphatase 3 (PFKFB3) [82]. In hypoxic lung carcinoma, tumour cells have been seen to evade CTL-mediated lysis, through autophagy induction, via a decrease in hypoxia-dependent induction of phosphorylated STAT3. Blocking hypoxia-induced autophagy in tumours restores cytotoxic T-cell activity and promotes tumour regression [83, 84]. Tumour cells can also escape NK cell-mediated immune surveillance by hypoxia-activated autophagy. In such case, granzyme B is selectively degraded upon autophagy activation, to inhibit NK cell-mediated target cell apoptosis. After target recognition by NK cells, the cytolytic effectors, like perforin 1 and granzyme B, enter the target cells and traffic to enlarged endosomes called “gigantosomes”. In normoxic cells, perforin forms pores in the gigantosome membrane, allowing granzyme B release and initiation of cell death. In hypoxic cells, excessive autophagy leads to fusion of gigantosomes with autophagosomes, and the subsequent formation of amphisomes, which contain granzyme B and perforin 1. The fusion of amphisomes with lysosomes triggers selective degradation of granzyme B, making hypoxic tumour cells less sensitive to NK cell-mediated killing [49]. However, autophagy also contributes to the expression of ICD-associated DAMPs. The hypoxic induction of autophagy enhances ICD through autophagy-mediated release of DAMPs. Other studies show that hypoxia prevents autophagy through inhibition of the mTOR pathway, making the exact role of HIFs in autophagy unclear [15, 85]. Autophagy can be stimulated by UPR induction, in response to endoplasmic reticulum (ER) stress and hypoxia. Depending on the type of stimulus and cellular damage, mTOR, UPR, and HIF-1 can all contribute to autophagy. This autophagic degradation of cellular components is attributed to hypoxic stress response [82]. Glucose deprivation as well as mitochondrial damage can also activate HIF-1, suggesting its feedback regulation by a set of interrelated signalling events, possibly through mTOR, UPR, and autophagy [82]. HIF-1 is identified to possess diverse regulatory roles in autophagy, with possible shared targets with mTOR. The autophagy pathways can, in turn, regulate HIF-1 stability. The authors Daskalaki et al. suggest that this reciprocal regulation of autophagy and HIF-1 activity can explain the opposing roles of autophagy activation in various human tumours [82].

#### Promotion of cancer cell stemness

**Fig. 4:**
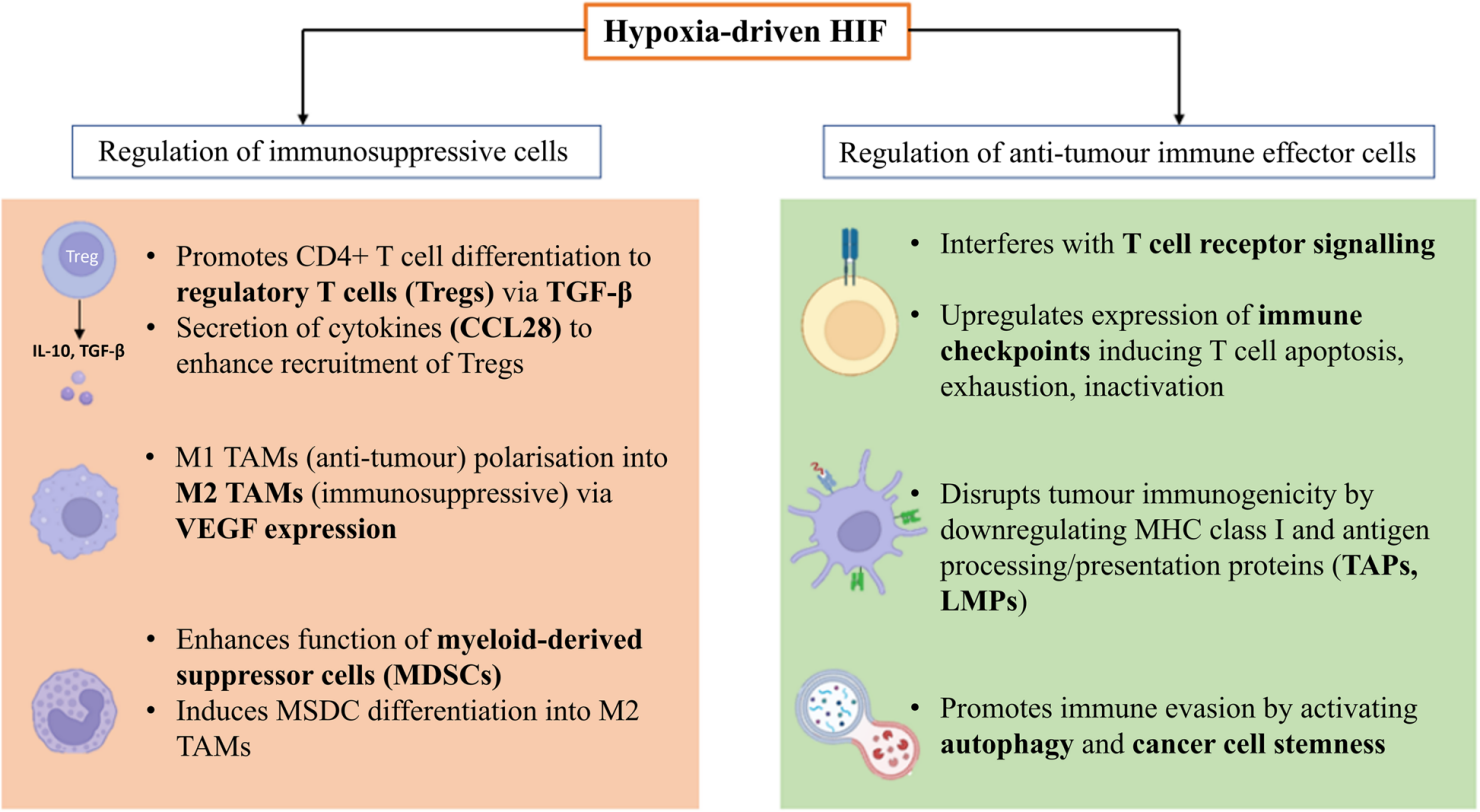
Summary of HIF-driven regulation of anti-tumour and pro-tumour immune cells

Cancer stem cells (CSCs) are found in small populations in the tumour. They are mainly associated with the immune silent phenotype in the hypoxic niche (Fig. 4). HIF stabilization is responsible for the adoption of stem cell properties in cancer cells, including multipotency and self-renewal capacity. During cancer progression, stemness of cancer cells is maintained by hypoxia, through different mechanisms, including enhancement of EMT, the transcriptional activation of stemness-related genes (Oct4, Nanog), and upregulation of signalling cascades responsible for maintaining stem-like features, such as BMP, Notch, WNT, JAK-STAT, and Sonic hedgehog (Shh), TGF-β, and IL-6/STAT3. VEGF has also been identified to play a role in stemness maintenance. It is produced by cancer stem cells under hypoxia. HIF-1 and HIF-2 are differentially expressed in CSCs but are both required for stemness, proliferation, and survival, although HIF-2 has been more frequently detected in CSCs [17]. Abundant research indicates the critical role of CSCs in suppressing anti-tumour immunity and allowing immune evasion of cancer cells. CSCs evade immune surveillance by exerting their effects on TAMs, DCs, MDSCs, Tregs, NK cells, and tumour-infiltrating lymphocytes [86]. Furthermore, PD-L1 has also been implicated in regulating the self-renewal of cancer stem cells, and its expression has been associated with stemness markers in many tumour types [87]. Yin, Shasha et al. demonstrated in their study that PD-L1-induced self-renewal capacity of endometrial cancer stem-like cells is dependent upon HIF-1α and HIF-2α activation, by the binding of HIFs to the PD-L1 promoter region. This suggests another consequence of HIF/PD-L1 interaction involved in maintaining the immunosuppressive nature of aggressive cancers through stemness [88].

### Targeting HIF to improve immunotherapeutic efficacy

Due to its multifaceted roles in regulating tumour immunity, the HIF signalling network has become a promising target for therapeutic interventions. A growing number of drugs targeting HIFs are being developed, which can block HIF directly by interfering with HIF dimerization, disrupting HIF mRNA or protein expression, promoting HIF degradation, preventing HIF/DNA binding and transcriptional activity, or targeting downstream targets of HIF signalling [89]. Hypoxia-active prodrugs or HAPs (bio-reductive drugs that are selectively activated under hypoxia) [90] have also gained increasing popularity, because they can specifically target hypoxic regions of tumour cells. Several HIF-1 inhibitors like acriflavine have been developed, to prevent HIF-1α/HIF-1β dimerization in prostate and colorectal cancer [91]. Anthracycline HIF-1 inhibitors can block HIF-1 transcriptional activity in hepatocellular carcinoma and prostate cancer [92]. Similarly, there are HIF-2 targeting inhibitors such as PT2385, which disrupts HIF-2α heterodimerization and inhibits HIF-2α target gene expression [93]. The orally administered MK-6482 prevents HIF-2 dimerization in renal cell carcinoma [94]. Table 1 describes some widely studied HIF inhibitors as well as drugs/therapeutic methods targeting other aspects of HIF signalling and regulation.

**Table 1:** HIF-1/2 and downstream target inhibitor drugs and clinical efficacy

Drug	Target	Mechanism of inhibition	Tumour	Ref.
Acriflavine	HIF-1	Prevents HIF-1α/HIF-1β dimerization	Prostate and colorectal cancer	[91, 95]
Anthracyclines	HIF-1	Inhibits HIF-1 transcriptional activity	Hepatocellular carcinoma, prostate cancer	[92]
Cardenolides			Breast cancer	[96]
PT2385	HIF-2	Disrupts HIF-2α/ARNT heterodimerization and inhibits HIF-2α target gene expression	Clear cell renal cell carcinoma	[93]
MK-6482				[94]
Benzopyranyl triazole	HIF-1	Increases HIF-1α hydroxylation and subsequent HIF-1α protein degradation	Used in combination with EGFR inhibitor gefitinib in lung and breast cancer	[97]
BIX01294 at low dosage			Hepatocellular carcinoma	[98]
IDF-1174	HIF-1	Increases HIF-1α protein degradation	Hepatocellular carcinoma, colorectal carcinoma	[99]
LBH589			Diffuse large B-cell lymphoma, Hodgkin lymphoma, multiple myeloma, hepatocellular carcinoma, pancreatic cancer and NSCLC	[99]
MPT0G157			Colorectal cancer	[100]
Vorinostat	HIF-1, HIF-2	Decreases HIF-1/2α translation/nuclear localization/stability, increases HIF-1/2 degradation	Hepatocellular carcinoma	[99]
Trastuzumab (Herceptin)	HIF-1 targets	Target oncogenic growth factor signalling pathways regulated by HIF-1	Breast cancer	[24]
Imatinib			Gastric gastrointestinal stromal tumours	
Galunisertib	HIF-1 targets	TGFβRI kinase inhibitor	Breast, colon, lung cancers, and hepatocellular carcinoma	[24]
Lenvatinib	HIF-1 targets	VEGF inhibitor	Hepatocellular carcinoma	[101]
SCH58261	HIF-1/2 targets	A2AR antagonist; inhibits immunosuppressive adenosine in TME	Head and neck squamous cell carcinoma	[102]

Since hypoxia exacerbates immunosuppression and immune evasion in solid tumours, it contributes significantly to immunotherapeutic resistance through the inhibition of effective anti-tumour immune responses. Hence, recent advances in cancer immunotherapy have explored combination therapies. Hypoxic treatments can be combined with standard immunotherapies, such as checkpoint inhibition, tumour vaccines using patient-specific neo-antigens, and CAR-T cells, to increase the sensitization of tumour cells to the treatment, enhance immunotherapeutic efficacy, and improve overall clinical outcomes. The synergistic effect of combination therapies targeting tumour immunity in conjunction with inhibition of hypoxic regulation of immune targets has yielded successful results in experimental models of many cancers. Several current and future clinical trials aim to further enhance these favourable therapeutic responses [5, 12]. Some of the most recent research involving HIF-targeting drugs and immunotherapy strategies acting in combination therapies, as well as their reported outcomes in tumour models, have been described in Table 2.

**Table 2:** Combination therapies tested in recent years

Drug	Mechanism of action	Combined with immunotherapy	Tumour	Cell line/model	Therapeutic results	Overall clinical outcome	Current investigations	Ref.
TH-302	Hypoxia-activated prodrug	Immune checkpoint blockade of PD-1 and CTLA-4	Prostate cancer	Transgenic adenocarcinoma of the mouse prostate-derived (TRAMP-derived) TRAMP-C2 cell line Male C57BL/6, Pb-Cre4, Ptenpc − / − Smad4pc − / − mice models	CD8 + T cells with increased granzyme B production, proliferation of CD44, and production of effector cytokines	Cured more than 80% of tumours TRAMP-C2 mice models Survival of mice with aggressive prostate adenocarcinoma significantly extended	Ongoing clinical trial (NCT03098160) being conducted to evaluate efficacy of TH-302 in combination with CTLA-4 blocker ipilimumab in melanoma, pancreatic, and prostate tumours	[103, 104]
PX-478	HIF-1α mRNA and protein translation inhibitor	Dendritic cell (DC) tumour vaccine	Breast cancer	4T1 cell line Female BALB/c mice bearing 4T1 tumours	Decreased hypoxia-induced HIF-1α protein levels and reduction in HIF-activated VEGF	Complete tumour regression in 50% mice with significant prolongation of survival	Ongoing studies on refining efficacy of DC vaccination especially in combination with HIF targeting drugs	[105] [106]
		Checkpoint blockade of PD-1	Non-small cell lung cancer (NSLC)	Human A549 and H1299 cell lines Female C57BL/6 mice	Inhibition of EMT-associated HIF-1α/LOXL2 axis, increased T-cell infiltration	Better sensitization of tumour to immune checkpoint blockade, marked delay in tumour growth, and prolonged survival	More clinical trials required to further verify the efficacy and safety of the HIF-1α inhibitor with anti-PD-1 blockade	[107]
Acriflavine	Inhibitor of HIF-1α/HIF-1β dimerization	Both TRP-2 peptide vaccine and anti-PD-1 blocking antibody	Melanoma and breast cancer	B16-F10 and 4T1 cell lines C57BL/6 and immunodeficient NOD SCID gamma mice	Inhibited melanoma tumour growth, increased infiltration of NK and CD8 + T cells into TME via release of CCL2 and CCL5 chemokines	Improved response to standard immunotherapy, better survival	Considerations for triple combination therapy	[108]
IDF-11774	HIF-1α inhibitor	Checkpoint blockade of PD-1	Prostate cancer	Murine prostate cell-derived xenograft (CDX) model set up in nude mice and BALB/c mice	Reduced MSDC and M2 macrophage population, increased T-cell infiltration	Augmented antitumor efficacy of PD-1 blockade, reduction in tumour volume	No data yet	[109]

## Discussion

Solid malignant tumours frequently develop hypoxic TMEs which impair anti-tumour immunity and compromise immunotherapeutic efficacy. The regulators of the majority of hypoxia-driven pathways are attractive targets that can be considered for reversing immunosuppression and restoring anti-tumour immunity. Several strategies targeting hypoxia have demonstrated synergy with existing immunotherapeutic approaches in exploiting the diverse HIF signalling network. Further clinical studies are needed to explore the most optimal combination strategies for tailored immunotherapy options in cancer patients. On the other hand, despite HIFs being confirmed to induce pro-tumourigenic events, not all hypoxia-mediated HIF activity is involved in immunosuppression. There is sufficient evidence of the contradictory roles of HIF in mediating tumour immunity. One mechanism as discussed earlier is the hypoxic activation of immunogenic cell death of tumours under ER stress [80]. Selective studies also suggest the role of HIF in enhancing the function of tumour-killing cells. For instance, Doedans et al. reported that elevated levels of HIF-1 and HIF-2 promoted the effector function of CD8 + T cells in tumours and infections based on microenvironmental cues [110]. In another study by Tyrakis et al., the HIF-1α/VHL axis was implicated in promoting CD8 + T-cell proliferation, differentiation, and antitumor activity through the regulation of the immune-metabolite S-2-hydroxyglutarate [111]. HIF-1 has also been found to contribute to NK cell priming and activation via regulation of the glycolytic rate [112]. These observations indicate possible underlying regulatory mechanisms of HIFs and other hypoxia-responsive molecules that are controlled by the type, localization, and action of TME cells and cytokines.

## Conclusion

Through a wide spectrum of direct and indirect regulation of target genes, HIFs play crucial roles in promoting tumour growth, proliferation, survival, and immune evasion by protecting cancer tissues from immune surveillance, upregulating the activity of immunosuppressive cells, and disrupting immune cytotoxicity against tumours. The HIF regulators are critical to our understanding of tumour cell adaptability and aggressiveness, as there are reports of the abnormal upregulation and stabilization of HIFs even in normoxic tumours. This has been observed in Kaposi sarcoma, breast, prostate, pancreatic, and kidney cancers [77, 78]. In a study on kidney cancer [113], TGF-β expression was observed to increase HIF-1α and HIF-2α under normoxic conditions. Another study on prostate cancer cell lines revealed that TGF-β induces HIF-1α, HIF-2α, and VEGF production under normoxia mediated by an interaction between Smad3 and HIF-2a in an HRE-dependent manner [114]. These observations may implicate factors like TGF-β as possible upregulators of HIF target genes in normoxic conditions and even more so in hypoxic environments, contributing to synergistic loops and promoting the development of aggressive and invasive cancer, regardless of oxygen tension [24]. These pathways may be important for future investigation of HIF signalling and subsequent targeting for modulation of tumour immunity.

## Data Availability

Not applicable.

## Abbreviations

ECM: Extracellular matrix
TME: Tumour microenvironment
TAM: Tumour-associated macrophage
DC: Dendritic cells
MDSC: Myeloid-derived suppressor cells
NK: Natural killer
TIME: Tumour immune microenvironment
CTL: Cytotoxic T lymphocyte
DC: Dendritic cell
APC: Antigen-presenting cell
FOXP3: Forkhead box P3
PMN-MDSC: Polymorphonuclear MDSC
M-MDSC: Monocytic MDSC
HIF: Hypoxia-inducible factor
PHD: Prolyl hydroxylase
HRE: Hypoxia-response elements
TGF: Tumour growth factor
NSCLC: Non-small cell lung carcinoma
VEGF: Vascular endothelial growth factor
IL: Interleukin
ROS: Reactive oxygen species
HLA: Human leukocyte antigen
DAMP: Damage-associated molecular patterns
UPR: Unfolded protein response
GLUT: Glucose transporters
HK: Hexokinase
LDHA: Lactate dehydrogenase
PGK1: Phosphoglycerate kinase 1
PDK1: Pyruvate dehydrogenase kinase 1
ENO1: Enolase 1
PFKFB3: 6-Phosphofructo-2-kinase/fructose-2,6-bisphosphatase 3
